# A Network Based Method for Analysis of lncRNA-Disease Associations and Prediction of lncRNAs Implicated in Diseases

**DOI:** 10.1371/journal.pone.0087797

**Published:** 2014-01-31

**Authors:** Xiaofei Yang, Lin Gao, Xingli Guo, Xinghua Shi, Hao Wu, Fei Song, Bingbo Wang

**Affiliations:** 1 School of Computer Science and Technology, Xidian University, Xi'an, Shaanxi, China; 2 Department of Bioinformatics and Genomics, College of Computing and Informatics, University of North Carolina at Charlotte, Charlotte, North Carolina, Unites States of America; University of Turin, Italy

## Abstract

Increasing evidence has indicated that long non-coding RNAs (lncRNAs) are implicated in and associated with many complex human diseases. Despite of the accumulation of lncRNA-disease associations, only a few studies had studied the roles of these associations in pathogenesis. In this paper, we investigated lncRNA-disease associations from a network view to understand the contribution of these lncRNAs to complex diseases. Specifically, we studied both the properties of the diseases in which the lncRNAs were implicated, and that of the lncRNAs associated with complex diseases. Regarding the fact that protein coding genes and lncRNAs are involved in human diseases, we constructed a coding-non-coding gene-disease bipartite network based on known associations between diseases and disease-causing genes. We then applied a propagation algorithm to uncover the hidden lncRNA-disease associations in this network. The algorithm was evaluated by leave-one-out cross validation on 103 diseases in which at least two genes were known to be involved, and achieved an AUC of 0.7881. Our algorithm successfully predicted 768 potential lncRNA-disease associations between 66 lncRNAs and 193 diseases. Furthermore, our results for Alzheimer's disease, pancreatic cancer, and gastric cancer were verified by other independent studies.

## Introduction

Long non-coding RNAs (lncRNAs) are similar to mRNAs in gene structure, with length greater than 200 nt [1]–[3]. LncRNAs play critical roles in many important biological processes such as chromatin modification [4], transcriptional and post-transcriptional regulation [4], and human diseases [2].

More and more studies have reported that mutated and dysfunctional lncRNAs are implicated in a broad range of human diseases. For example, Pasmant et al. [5] performed a GWAS and identified that *ANRIL* was significantly associated with coronary disease, type 2 diabetes, and many types of cancers. *HOTAIR* was increased from 100 to approximately 2,000-fold in breast cancer metastases using quantitative PCR [6]. *MALAT-1* was significantly associated with metastasis in NSCLC patients by quantitative RT-PCR [7]. With regard to Alzheimer's disease, *BCAE1-AS* was shown to have a key role in regulating *BACE1* and in driving pathology [8]. Cui et al. [9] found that the expression of *PlncRNA-1* was significantly higher in prostate cancer cells. Therefore, it is necessary to analyze the available lncRNA-disease associations and predict potential lncRNA-disease associations in human. Such studies will help us understand the molecular mechanisms of human diseases and identify biomarkers for disease diagnosis, treatment, and prevention at lncRNA level [10].

Chen et al. [10] reported a LncRNADisease database that includes approximately 480 entries of experimentally supported associations between 166 diseases and 118 lncRNAs. Moreover, we have manually collected 380 lncRNA-disease associations between 226 lncRNAs and 145 diseases by literature mining. By integrating these two data sets, we obtained 578 lncRNA-disease associations between 295 lncRNAs and 214 diseases. These data were analyzed in a network view and used to predict lncRNA-disease associations.

**Figure 1:**
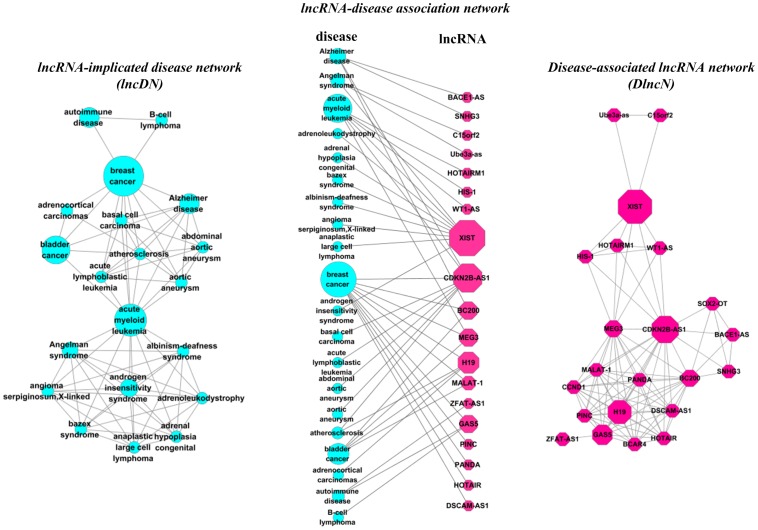
Construction of the lncRNA-disease bipartite network. *(Center)* A subnetwork of the full lncRNA-disease association network (Figure S1), where the blue circles and red hexagons correspond to diseases and lncRNAs, respectively. A link is placed between a disease and a lncRNA if mutations or dysfunctions in that lncRNA lead to the specific disease. The size of a blue circle is proportional to the number of lncRNAs participating in the corresponding disease. The size of a red hexagon is proportional to the number of diseases associated with the corresponding lncRNA. *(Left)* The lncDN projection of the center graph, in which two diseases are connected if there is a lncRNA implicated in both diseases. *(Right)* The DlncN projection of the center graph where two lncRNAs are connected if they are involved in the same disease.

**Figure 2:**
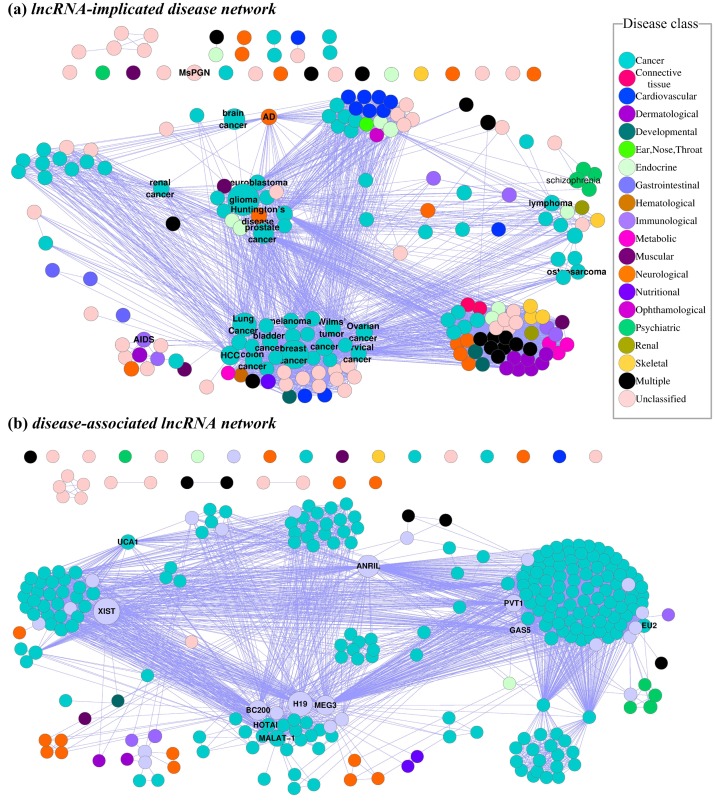
The lncDN and DlncN. (a) In lncDN, each node corresponds to a distinct disease, colored based on the disease class [26] to which it belongs. The names of 20 disease classes are shown on the right panel. A link between two diseases exists if they share at least one implicated lncRNA. The size of the node is proportional to the degree of the node in lncRNA-disease association network. We label the diseases associated with more than five lncRNAs by their names. (b) In DlncN, each node is a lncRNA, with two lncRNAs being connected if they are implicated in the same disease. The size of each node is proportional to the number of diseases in which the lncRNA is implicated. The color of a node is based on the class of diseases in which the corresponding lncRNA implicated. Nodes are light purple if the corresponding lncRNAs are associated with more than one disease class. We label the lncRNAs implicated in more than five diseases by their names.

In this paper, based on the available lncRNA-disease associations, a lncRNA-disease association network was constructed. From the constructed network, two relevant biological networks “lncRNA-implicated disease network” (lncDN) and “disease-associated lncRNA network” (DlncN) were derived, as shown in Figure 1. In lncDN, a node represented a disease, and a link between two nodes indicated that the two corresponding diseases shared at least one lncRNA as their disease-causing lncRNA (Figure 1 and Figure 2-(a)). In DlncN, a node represented a lncRNA, and a link between two nodes represented the fact that the two corresponding lncRNAs were implicated in at least one common disease (Figure 1 and Figure 2-(b)). The known lncRNA-disease associations were represented in a single network framework, and the network topological properties were analyzed to help us investigate all of these associations. Furthermore, a propagation algorithm was applied to predict potential lncRNA-disease associations on the lncRNA-disease association network. In addition, a coding-non-coding gene-disease bipartite network was constructed by integrating coding gene-disease associations obtained from OMIM [11] with lncRNA-disease associations. To achieve better prediction performance, the propagation algorithm was applied to rank the potential gene-disease pairs for all the diseases on the coding-non-coding gene-disease bipartite network. In the Leave-One-Out Cross-Validation (LOOCV) procedure, our method achieved a reliable Area Under Curve (AUC) of 0.7881. We then employed our method to the study of three multi-factorial diseases, Alzheimer's disease, pancreatic cancer and gastric cancer, and provided suggestions of novel disease-causing lncRNAs for further study.

## Materials and Methods

### Data Sources

The 480 lncRNA-disease associations were downloaded from LncRNADisease database [10], including 118 lncRNAs and 166 diseases. Note that many other lncRNA-disease associations have been reported in the literature, but have not been included in the LncRNADisease database yet. Hence, we retrieved literature from PubMed (http://www.ncbi.nlm.nih.gov/pubmed), employing the key words ‘lncRNA and disease’, ‘lncRNA and cancer’, ‘long non-coding RNA and disease’, ‘long non-coding RNA and cancer’, ‘lincRNA and disease’ or ‘lincRNA and cancer’, and manually extracted 129 articles that reported lncRNA-disease associations. In this way, we collected an additional 380 lncRNA-disease associations between 226 lncRNAs and 145 diseases by literature mining. Integrating these two data sets from both LncRNADisease database and literature search, we finally obtained 578 associations between 295 lncRNAs and 214 diseases. All of these 578 lncRNA-disease associations were then merged into a lncRNA-disease association network.

Of the 214 diseases, 160 diseases and their causative genes could be found using their MIM number in OMIM database [11]. In total, we downloaded 801 disease genes for these 160 diseases from OMIM database. Such data resulted in 980 protein-coding gene-disease associations that were used in our method.

Integrating lncRNA-disease associations and protein-coding gene-disease associations obtained above, we obtained 1558 coding-non-coding gene-disease associations between 1096 genes (295 lncRNAs and 801 protein-coding genes) and 214 diseases. These associations were used to construct the coding-non-coding genes-disease bipartite network.

## Methods

Given a bipartite network 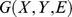, 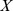 and 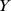 were two disjoint node sets, 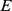 was the edge set in which the element represented the edge connecting the node from 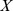 and the node from 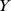. The bipartite network could be viewed in one-mode projection onto 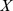 and one-mode projection onto 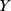, called 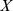 projection and 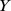 projection respectively. The 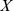 projection of 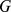 was a network in which nodes were from 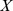, and the edge indicated that the connected nodes were associated with at least one same node from 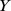. Similarly, the 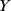 projection of 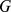 was a network in which nodes were from 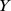, and the edge indicated that the connected nodes were associated with at least one same node from 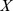. With regard to the lncRNA-disease association network, which could be claimed as a bipartite network, lncDN and DlncN were the disease projection and lncRNA projection of the lncRNA-disease association network. The properties of these two projections were analyzed in the “Results” section. It was found that the lncDN could reflect the relationships between any two diseases at the lncRNA level and that DlncN could reflect the relationships between any two lncRNAs at the disease level. Moreover, we tried to exploit these relationships to predict the hidden lncRNA-disease associations. For better performance, both protein-coding genes and lncRNAs that were implicated in diseases were considered together. As a result, a coding-non-coding gene-disease bipartite network was constructed to reflect the associations between diseases and all the disease-causing genes (i.e. protein-coding genes or lncRNAs). The resource-allocation process [12], as one of the best weighting methods for one-mode projection of a bipartite network, was used to weight the gene projection of the coding-non-coding gene-disease bipartite network. Then a propagation algorithm was applied to compute the association score for each gene that was used to measure how much the gene could be implicated in a disease on the weighted gene projection. For a disease 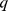, every gene had its initial information. Our propagation algorithm could be assumed as a process where genes pumped their initial information to their neighbors, and every gene propagated the information received in the previous iteration to other genes via edges in gene projection.

Next, we illustrated the principle of the resource-allocation process, and then provided the propagation algorithm to compute the score of genes with respect to a specific disease.

### Principle of the resource-allocation process

**Figure 3:**
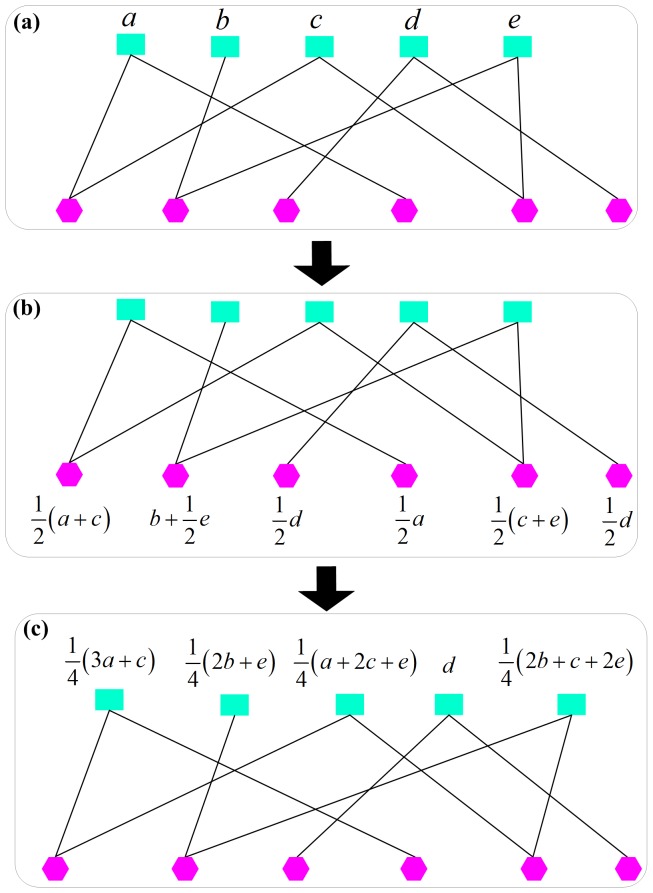
Principle of the resource-allocation process in a bipartite network. The green rectangles represent 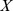 nodes and red hexagons represent 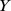 nodes. The whole process consists of two steps: First, the resource flows from 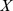 to 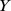 (a
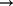
b), and then returns to 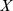 (b
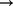
c).

We divided the nodes of a bipartite network into two sets 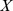 and 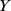, and only the connections between two nodes in different sets are allowed. The resource-allocation process is one of the best weighting method for one-mode projection of a bipartite network [12]. This process was illustrated in Figure 3 and included the following two steps. First, we allocated resources from 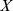 to 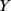. Second, we then allocated resources from 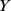 back to 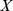. The initial resource of five nodes was 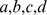 and 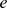 in set 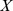. These two steps of the resource-allocation process were merged into one, and the final resource of 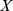 nodes denoted by 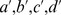 and 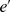, could be written as:
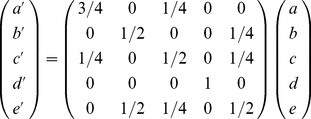
(1)The 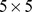 matrix, 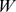, represented the weighted 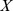 projection. The element 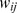 represented the fraction of resource that the 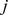-th 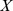 node transferred to 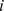-th 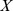 node, and could be considered as the importance of node 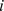 on node 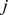 [12].

For a bipartite network 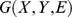, and 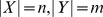, 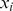 was the 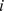-th 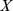 node and 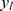 was the 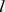-th 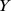 node. 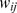 could be calculated as follows [12]:
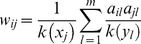
(2)where 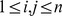, 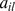 was the 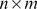 adjacent matrix of 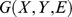, and 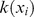 was the degree of 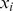.

### The propagation algorithm

The coding-non-coding gene-disease bipartite network was denoted by 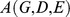, where 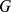 was the node set of the genes, 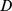 was the node set of the diseases, and 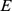 was the edge set. The weighted gene projection of 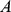 was denoted by 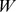, where 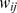 was calculated by Formula (2) and represented the importance of gene 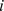 on gene 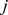 in terms of their association with disease.

Our propagation algorithm was based on a semi-supervised learning algorithm [13], which had been previously used to prioritize protein-coding genes implicated in human diseases [14] and annotate functions of lncRNAs [15]. The input of the algorithm included 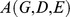, a query disease 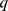, and 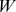. For disease 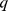, every gene had its own initial information. If a gene was connected with 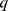 in the coding-non-coding gene-disease bipartite network, the initial information was one; otherwise the initial information was zero. For a given disease 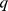, the score vector 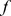 of genes represented the association scores of genes with 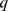, which was computed by an iterative algorithm. The genes were ranked for 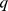 by the final score vector. Of all the genes not associated with disease 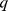, the top 1% ranked genes were considered as the predicted genes. The score vector 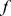 was defined as:
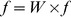
(3)An iterative process [14] was applied to compute the score vector in Formula (3). Considering the initial information on the genes for the given disease, the score vector 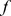 was computed iteratively as follows,
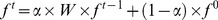
(4)In Formula (4), the score vector was initialized as 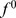 by the initial information on genes. The parameter 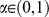 gave the relative importance between the contributed information of other genes and the initial information of itself. The final score vector with respect to disease 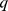 was determined by both the information on other genes and its initial information. The iterative computation was controlled by the mean score deviation of the two neighboring score vector. All the testes on the real data and random data had shown that the iterative computation converges eventually (Table S1).

## Results

### Properties of lncRNA-disease association network

The available lncRNA-disease associations were modeled as a bipartite network, and a subnetwork of this network was shown in Figure 1. In this bipartite network, one node set corresponded to the disease set; the other set corresponded to the lncRNA set. A lncRNA and a disease were connected by a link if the lncRNA was associated with the disease. The constructed bipartite network contained 578 edges between 295 lncRNA nodes and 214 disease nodes.

The degree distribution of the full lncRNA-disease association network (Figure S1) closely followed a power-law distribution (Figure S2-(a)). We also analyzed the degree of disease nodes and that of lncRNA nodes separately. The degree of a disease node, which meant the number of lncRNAs associated with the disease, was denoted by 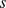 and had a broad distribution (Figure S2-(b)). These results indicated that most disorders were associated with a small number of lncRNAs, whereas a handful of diseases, such as breast cancer and lung cancer, were related to a large number of lncRNAs. For example, 41 lncRNAs were involved in breast cancer (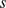 = 41), 18 lncRNAs were related with prostate cancer (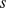 = 18), and 28 lncRNAs were involved in lung cancer (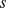 = 28). The degree of a lncRNA node, i.e. the number of diseases associated with the lncRNA, was denoted by 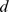, and had a broad distribution as well (Figure S2-(c)). This indicated that many lncRNAs were related to a few diseases whereas a small number of lncRNAs could be related to dozens of diseases. For example, *XIST* (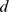 = 50) was associated with 50 diseases, including 40 skin diseases [16] and certain types of cancers such as testicular cancer [17] and breast cancer [18]. *H19* (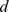 = 39) was associated with 39 diseases, including Beckwith-Wiedemann syndrome [19], Silver-Russell syndrome [20], [21] and many types of cancer [22]. *MEG3* (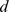 = 23) was associated with 23 diseases, including breast cancer [23], bladder cancer [23], glioma [24], and Wilms' tumor [25], etc. These lncRNAs represented major hubs in DlncN (Figure 2-(b)).

### Network analysis of lncDN and DlncN

We performed a network analysis of lncDN and DlncN to help us understand the lncRNA-disease associations. Two biologically relevant network projections, lncDN and DlncN, were generated (Figure 2) based on the lncRNA-disease association network. Specifically, lncDN provided a disease centered view of the lncRNA-disease association network (Figure 2-(a)). DlncN was complementary to lncDN and offered a lncRNA centered view of the lncRNA-disease association network (Figure 2-(b)). Especially, the links between two lncRNAs in DlncN signified the disease phenotypic associations, which might be a measure of their functional correlations and could be used in future studies.

#### Degree distributions of lncDN and DlncN

The degree distribution of the lncDN was investigated (Figure S3-(a)). The results showed that most disorders linked to only a few other diseases, whereas only few disorders represented hubs that were connected to a large number of distinct disorders. Such hub disorders included breast cancer (linked to 150 other disorders, i.e. 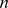 = 150, here 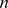 meant the degree of a node in lncDN), prostate cancer (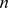 = 144), and lung cancer (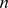 = 73). The degree distribution of the DlncN (Figure S3-(b)) was similar to that of lncDN. We could see that the degrees of most lncRNAs were small, whereas a few lncRNAs linked to a large number of lncRNAs. For example, *MEG3* linked to 196 other lncRNAs, *ANRIL* linked to 166 other lncRNAs, and *PVT1* linked to 162 other lncRNAs. These highly connected lncRNAs represented hubs in DlncN which connected to a large number of diseases in lncRNA-disease association network. We concluded that the degree distributions of both lncDN and DlncN networks closely followed a power-law distribution, despite of the incompleteness and false positive rate of the known lncRNA-disease associations.

#### Comparison of lncDN and DlncN with random networks

In lncDN, there were 3061 links among 214 individual diseases. Of the 214 diseases, 197 had at least one link to other diseases and 182 diseases formed a giant connected component. In DlncN, there were 6989 links among 295 lncRNAs. Of the 295 lncRNAs, 276 had at least one link to other lncRNAs and 265 lncRNAs formed a giant connected component.

We randomly shuffled the lncRNA-disease association network for 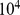 times, while keeping the degree of each lncRNA and each disease in the bipartite network unchanged [26]. We constructed the corresponding r-lncDN and r-DlncN respectively for the disease and lncRNA centered view of the randomized lncRNA-disease association network. Comparing lncDN and DlncN with r-lncDN and r-DlncN, respectively, we found that the topology property of the two generated networks, lncDN and DlncN, deviated from random. The average size of the giant connected components of 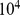 r-lncDNs was 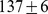, which was significantly smaller than 182 (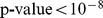, *z*-test), the actual size of the giant connected component of lncDN. Similarly, the average size of the giant connected components of 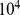 r-DlncNs was 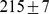, which was significantly smaller than 265 (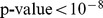, *z*-test), the actual size of the giant connected component of DlncN. Considering disease classes as defined in the Goh et al.'s study [26], we found that diseases (lncRNAs) were more likely to be linked to the diseases in the same class in the actual networks. For example, in the lncDN, there were 806 links between diseases of the same class, a two-fold enrichment with respect to 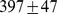 links obtained between the same set of nodes in the randomized networks. These differences suggested important pathophysiological clustering of diseases and disease associated lncRNAs.

#### Clustering coefficients of lncDN and DlncN

To further address the topological properties of lncDN and DlncN, we calculated the average clustering coefficient, a measure of the tendency of nodes in a network to form clusters or groups [27], by NetworkAnalyzer [28], a plugin of cytoscape software [29]. We found that the average clustering coefficients of nodes in both networks approximately diminished when the degree of node increased (Figure S4), indicating that nodes with high degrees tended to be hub nodes in both networks. In addition, we calculated the clustering coefficients of lncDN as the average of the clustering coefficient of all the vertices in lncDN [30], and the clustering coefficients of 550 randomly generated networks with the same degree sequence as lncDN [31]. The average clustering coefficient of the randomized networks was 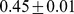, which was significantly smaller than 0.81 (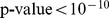, *z*-test), the clustering coefficient of lncDN. Likewise, we generated 550 randomized networks with the same degree sequence as DlncN. The average clustering coefficient of the 550 randomized networks was 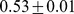, which was significantly smaller than 0.91 (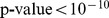, *z*-test),the clustering coefficient of DlncN. These results indicated that lncDN and DlncN revealed obvious community structure. Therefore, in the following section, we would like to analyze the modules of lncDN and DlncN.

#### Modules of lncDN and DlncN

We clustered the lncDN and DlncN by MINE (http://apps.cytoscape.org/apps/mine), a plugin of cytoscape software [29]. As a result, we obtained 14 modules of lncDN and 19 modules of DlncN. The size of each module had a board distribution (Figure S5).

Although the lncDN layout was generated without any knowledge on the disease classes, the resulted network was visibly clustered according to major disease classes (Figure 2-(a)). For example, most (seven in 11) diseases that belonged to cardiovascular were associated with *ANRIL* and were clustered together. Most (seven in eight) dermatological diseases were associated with *XIST* and were also clustered into one cluster. However, some lncRNAs might be of special importance as they were implicated in different cancers which were not clustered into a single cluster. For example, *ANRIL* was associated with 14 types of cancer and *MEG3* was associated with 18 types of cancer. These observations suggested the complexity and heterogeneity of different types of cancers.

In DlncN, lncRNA nodes were colored based on the class of diseases in which these lncRNAs were implicated. Nodes were light purple if the corresponding lncRNAs were associated with more than one disease class (Figure 2-(b)). We found that most lncRNAs were only implicated in certain type of cancers, and they were mostly clustered into one module. For example, 17 lncRNAs were only related to brain cancer, 22 lncRNAs were only related to breast cancer, and 84 lncRNAs were only related to glioma. However, the major hubs were related to more than one disease class, such as *XIST* that was related to 12 disease classes, *H19* was related to seven disease classes, *ANRIL* was related to six disease classes, and *MEG3* was related to four disease classes. These results were consistent with the fact that many lncRNAs exhibited tissue-specific expression [32] and that a few lncRNA were expressed across many tissues, such as *MEG3*, *XIST*, and *H19* [33].

### Prediction of lncRNAs implicated in diseases

We applied the propagation algorithm to predict the candidate gene-disease associations on the coding-non-coding gene-disease bipartite network. In this algorithm, there were two parameters to be tuned: 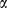 and 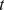. The parameter 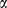 gave the relative importance between the information that other genes contribute and the initial information. This parameter was tuned by LOOCV tests and “0.618” was chosen as our 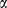 based on this procedure. The parameter 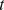 represented the number of iterations. The iterative computation would stop if the mean square deviation of the coding-non-coding gene-disease association score matrix between the 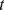-th iteration and the 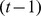-th iteration was not greater than 0.00001. With these two parameters, our algorithm ranked 2139 potential gene-disease pairs (768 lncRNA-disease pairs and 1371 coding gene-disease pairs) within top 1% for all the diseases. In the LOOCV procedure, our method achieved an AUC of 0.7881.

### Robustness of our bipartite network

We tested the robustness of the coding-non-coding gene-disease bipartite network using the method of Multiple Survival Screening (MSS) [34], which was introduced to test the robustness of cancer causing genes by re-sampling experiments. Here, we performed 1000 times of re-sampling of our coding-non-coding gene-disease associations to predict the potential gene-disease associations. In each re-sampling experiment, we randomly removed 10% edges from the coding-non-coding gene-disease bipartite network, and then applied the propagation algorithm to predict the potential gene-disease associations on the remaining bipartite network with 90% edges.

**Figure 4:**
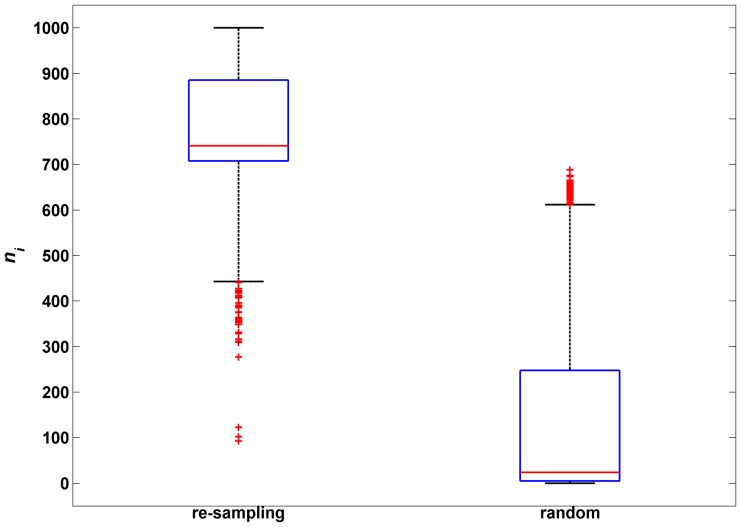
Comparison between re-sampling and random experiments to investigate the robustness of the bipartite network. Two box graphs represent the re-sampling experiments (left) and the random experiments (right), 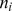 means the time of predicted association 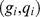 can be also predicted in re-sampling experiments and random experiments.

If a gene 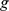 was ranked within top 1% among all the genes according to the score vector for a given disease 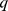, then the gene 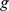 was predicted to be associated with the disease 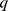, i.e. the gene-disease pair 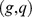 was considered as a predicted association. Applying the propagation algorithm on the coding-non-coding gene-disease bipartite network, we obtained 2139 predicted associations. For a predicted association 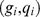 (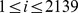), if the rank of 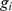 was within top 1% in a re-sampling experiment, then 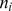 was increased by one. A vector 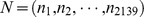 was obtained, where 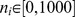, meant the times of the predicted association 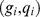 could be also predicted in 1000 re-sampling experiments. Furthermore, we performed 1000 times of random experiments. In each experiment, we randomly shuffled the coding non-coding gene-disease bipartite network, while keeping the degree of each gene and each disease in the bipartite network unchanged as above, and then applied the propagation algorithm to the randomized network. Similarly, a vector 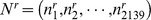
(*r* represented random) was obtained. We found that 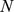 was significantly larger than 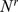 (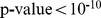, *z*-test), with most of 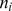
s larger than 700, and most of 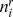
s smaller than 250 (Figure 4). These findings suggested that even the 10% edges of the coding-non-coding gene-disease bipartite network were deleted, the predictive results were still stable. Therefore, our coding-non-coding gene-disease bipartite network was sufficiently robust to predict potential coding or non-coding gene disease associations.

### Leave-one-out cross-validation tests

To evaluate the power of our method, we applied the LOOCV procedure. In each test of LOOCV, a single gene-disease association was removed from the coding-non-coding gene-disease bipartite network, and the method was evaluated by its success in reconstructing the hidden association. If the degree of gene or disease node in the removed gene-disease association was exactly one, then the gene or disease would be an isolated node. An isolated node in the propagation algorithm could not get any information, so we removed the nodes whose degree was one in LOOCV. Finally, we kept 532 links between 103 diseases and 163 genes (mapped to 44 lncRNAs and 119 protein-coding genes) that were to be used in LOOCV. To the best of our knowledge, this was the first work of predicting potential lncRNA-disease associations in a network view, therefore, no previous methods could be directly compared with our method. We would compare the predictive performance of the propagation algorithm on different networks.

**Figure 5:**
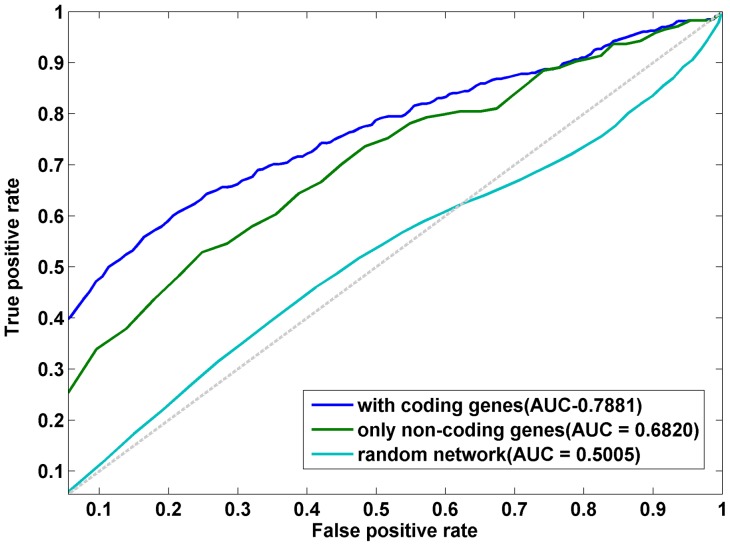
A comparison between the performance of our propagation algorithm on coding-non-coding gene-disease bipartite network and that on lncRNA-disease association network. The blue line represents the ROC curve of taking LOOCV over coding-non-coding gene-disease bipartite network, and an AUC of 0.7881 was obtained. The cyan line represents the ROC curve of taking LOOCV over lncRNA-disease association network, and an AUC of 0.6820 was obtained. The light blue line represents the ROC curve of taking LOOCV over random networks, and an average AUC of 0.5005 was obtained.

The receiver operating characteristics (ROC) curve was used to measure the performance of our method, which plotted the true positive rate (TPR) versus the false positive rate (FPR) at different rank thresholds. In LOOCV, for a rank threshold 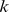 (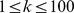), TPR meant the percentage of the leave-out associations obtaining the rank within top 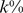; FPR meant the percentage of unassociated gene-disease pairs obtaining the rank within top 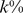. When the rank threshold was varied between 1 and 100, the corresponding TPR and FPR were obtained. In this way, the ROC curve could be plotted, and the AUC could be calculated. Following this procedure, we performed LOOCV over lncRNA-disease association network, and achieved an AUC of 0.6820. The ROC was shown in Figure 5.

Aiming at improving the performance of our method, we integrated the protein coding gene-disease associations with lncRNA-disease associations to construct the coding-non-coding gene-disease bipartite network. Here, we also performed LOOCV procedure over coding-non-coding gene-disease bipartite network and obtained an AUC of 0.7881. The ROC was shown in Figure 5. Clearly, the integration of protein coding gene-disease associations could improve the performance of our method. One reason of the improvement was that the number of edges in the bipartite network was increased by the integration. Therefore, potential genes could get more information from other genes and diseases in propagation and could be better predicted. The better performance might be also attributed to the fact that coding and non-coding genes were cooperated in human diseases. Therefore, the performance of our method would be further improved after obtaining more known lncRNA-disease associations, and more associations between coding genes and non-coding genes.

Moreover, we performed the LOOCV procedure over 50 random networks. The mean FPR and mean TPR were used to plot the ROC curve (Figure 5), and we achieved an AUC of 0.5005, smaller than AUCs of other two cases. This indicated that our coding-non-coding gene-disease bipartite network could reflect some mechanisms of human complex diseases, and our method could discover potential lncRNA-disease associations.

### Case study

For each disease, all the genes (including coding and non-coding genes) were ranked according to their association scores with the disease. The genes ranked within top 10 (this was a user-defined threshold, and 10 was used here) were considered as the potential genes involved in the given disease. For all the 214 diseases in the coding-non-coding gene-disease bipartite graph, we uncovered 768 novel lncRNA-disease associations between 66 lncRNAs and 193 diseases.

To further demonstrate the power of our method, we examined the results for three multifactorial diseases (i.e. Alzheimer's disease (MIM: 176807), pancreatic cancer (MIM: 260350) and gastric cancer (MIM: 137215)) as case studies. For each case, the top 10 genes including protein-coding genes and lncRNAs were listed in Table 1.

**Table 1:** The top-10 ranked genes for three case studies.

Alzheimer's disease					
gene	ACC/MIM	Rank	gene	ACC/MIM	Rank
COL4A2	120090	1	IL1RN	147679	6
*H19*	*NR_002196*	2	EPO	133170	7
*PVT1*	*NR_003367*	3	SOD2	147460	8
ALOX5AP	603700	4	VEGF	192240	9
PON1	168820	5	F2	176930	10

Pancreatic cancer					
gene	ACC/MIM	Rank	gene	ACC/MIM	Rank
*H19*	*NR_002196*	1	FGFR3	134934	6
*ANRIL*	*NR_003529*	2	*UCA1*	*NR_015379*	7
*BC200*	*NR_001568*	3	PIK3CA	171834	8
*MEG3*	*NR_002766*	4	CDH1	192090	9
*XIST*	*NR_001564*	5	*SRA*	*AF092038*	10

Gastric cancer					
gene	ACC/MIM	Rank	gene	ACC/MIM	Rank
*XIST*	*NR_001564*	1	*ANRIL*	*NR_003529*	6
*MALAT-1*	*NR_002819*	2	TP53	191170	7
*MEG3*	*NR_002766*	3	FGFR3	134934	8
*PVT1*	*NR_003367*	4	PTEN	601728	9
BRCA2	600185	5	RAD54L	603615	10

In this table, the susceptibility genes (protein-coding genes and lncRNAs) for three case studies including Alzheimer's disease, Pancreatic cancer and Gastric cancer were listed. The genes in italic were lncRNAs and the others were protein-coding genes.

#### Results for Alzheimer's disease

Alzheimer's disease (AD) is the most common form of dementia in the elderly [35] and it is characterized by slow progressive loss of memory, cognitive abilities, and intellectual functions [36]. Currently, it has been reported that 23 genes including 6 lncRNAs and 17 protein-coding genes are associated with AD. The association scores of these 23 genes were higher than unassociated genes. In the top-10 ranked genes unassociated with AD, we found that the rank of lncRNA *H19* was two, and the rank of lncRNA *PVT1* was three. *H19* had been associated with glioblastoma [37] and *PVT1* had been associated with glioma [24]. Both glioblastoma and glioma were brain or neuron related diseases and AD was described as a neurological disease. All these suggested the relationship between these two lncRNAs and AD.

#### Results for Pancreatic cancer

Pancreatic cancer has a high mortality rate and the 5-year relative survival rate is less than 5% [38]. It has been previously shown that 18 genes including 5 lncRNAs and 13 protein-coding genes are implicated in pancreatic cancer. The association scores of these 18 genes were also higher than unassociated genes. In the top-10 ranked genes unassociated with pancreatic cancer, we found that the rank of *ANRIL* was two. Pasmant et al. [5] confirmed the pivotal role of *ANRIL* in regulation of *CDKN2A/B* expression through a *cis*-acting mechanism in mice and *ANRIL* implicated in proliferation and senescence. Furthermore, the association of *CDKN2A* (MIM:600160) with pancreatic cancer had been curated in OMIM [11]. The rank of *UCA1* was seven, and Kaneko et al. [39] showed that *UCA1* and *BMF* were upregulated in gallbladder epithelia of children with pancreaticobiliary malfunction. Therefore, our results suggested that *UCA1* might be associated with pancreatic disease.

#### Results for Gastric cancer

Gastric cancer is a high morbidity cancer and has varied morbidities in different populations [40]. It has been presented that 15 genes including 4 lncRNAs and 11 protein-coding genes are implicated in gastric cancer. The association scores of these 15 genes were higher than unassociated genes. In potential genes implicated in gastric cancer, the rank of *XIST* was one. Weakley et al.'s study [41] showed that *XIST* was differentially expressed in preneoplastic cells located in gastric fundus that could lead to gastric cancer. The rank of *MEG3* was three, and *MEG3* was reported to function as a novel lncRNA tumor suppressor [42].

## Discussion

The lncRNA-disease association network was constructed, from which two relevant networks, lncDN and DlncN, were generated accordingly. These networks provided a unified framework of all known lncRNA and disease associations and a new network view for the study of the lncRNA-disease associations. The detailed lncRNA-disease association network (Figure S1) showed all the known lncRNA-disease associations. Furthermore, a computational iterative algorithm was applied to mine the hidden lncRNA-disease associations. The results showed that our method could provide insightful suggestions of lncRNA implicated in diseases.

Our method had some limitations that should be acknowledged. First, the analysis of the function of lncRNAs on a whole genome-wide scale was limited due to the diversity, lack of knowledge and specificity of expression of lncRNAs, and the lack of lncRNA functional annotation. Second, the shortage of lncRNA-disease associations limited the analysis of the mechanism of lncRNAs implicated in disease on a larger network. Finally, due to the lack of interactions and similarities between non-coding genes and protein coding genes, it was insufficient in biological meaning to replace the gene similarity matrix in Formula (4) by the weighted gene projection 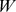.

## Supporting Information

Table S1
**20 tests on random networks to show that the propagation method converges.** We did 20 tests. In test 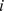 (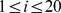), we applied the propagation method on 100 random networks with the mean square deviation threshold between 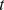-th iteration and 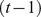-th iteration being 10E-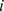. The average iteration times were calculated and listed.(XLSX)

Figure S1 **Bipartite-graph representation of the lncRNA-disease association network.** A disease (circle) and a lncRNA (hexagons) are connected if the lncRNA is implicated in the disease. The size of a node is proportional to the degree of the node. The color of a disease node (circle) represents the class which it belongs. The names of 20 disease classes are shown on the right panel. The color of a lncRNA node (hexagons) is based on the class of diseases in which the corresponding lncRNA implicated. LncRNA Nodes are light purple if the corresponding lncRNAs are associated with more than one disease class. We label the diseases (lncRNAs) associated with more than five lncRNAs (diseases) by their names.
(TIF)

Figure S2
**Degree distribution of full lncRNA-disease association network.** (a) The degree distribution of the full lncRNA-disease association network. It closely follows a power-law distribution. Here, 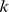 represents degree, 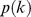 denotes the fraction of nodes with a given degree 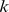. (b) Degree distribution of disease nodes in lncRNA-disease association network. (c) Degree distribution of lncRNA nodes in lncRNA-disease association network.(TIF)

Figure S3
**Degree distribution of lncDN and DlncN.** (a) Degree distribution of lncDN. It closely follows a power-law distribution. Here, 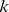 represents degree, 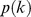 denotes the fraction of nodes with a degree 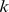. (b) Degree distribution of DlncN. It closely follows a power-law distribution. Here, 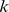 represents degree, 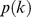 denotes the fraction of nodes with a degree 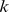.(TIF)

Figure S4 **Degree distributions of average clustering coefficients of nodes in lncDN and DlncN.** (a) Degree distribution of average clustering coefficients of nodes in lncDN. (b) Degree distribution of average clustering coefficients of nodes in DlncN. Both distributions are closely following a power-law distribution.
(TIF)

Figure S5 **Distribution of module sizes in lncDN and DlncN.** (a) The module sizes of 14 modules in lncDN. (b) The module sizes of 19 modules in DlncN.
(TIF)
