# Supplementary figures and images for: A Network Based Method for Analysis of lncRNA-Disease Associations and Prediction of lncRNAs Implicated in Diseases
Source: PLoS One. 2014 Jan 31;9(1):e87797. doi: 10.1371/journal.pone.0087797 (PMC3909255; doi:10.1371/journal.pone.0087797)

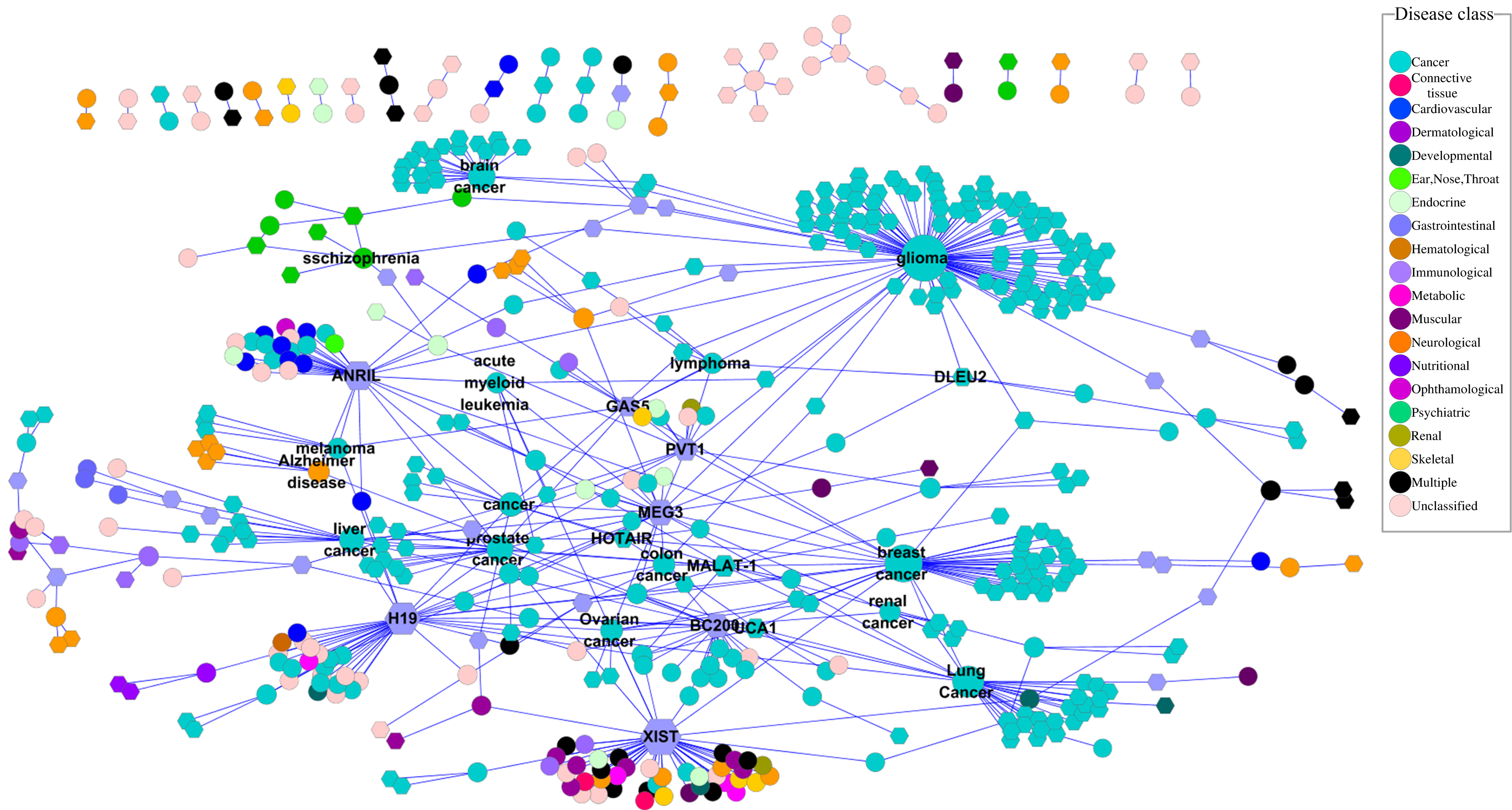

Supplement: Figure S1 — Bipartite-graph representation of the lncRNA-disease association network. A disease (circle) and a lncRNA (hexagons) are connected if the lncRNA is implicated in the disease. The size of a node is proportional to the degree of the node. The color of a disease node (circle) represents the class which it belongs. The names of 20 disease classes are shown on the right panel. The color of a lncRNA node (hexagons) is based on the class of diseases in which the corresponding lncRNA implicated. LncRNA Nodes are light purple if the corresponding lncRNAs are associated with more than one disease class. We label the diseases (lncRNAs) associated with more than five lncRNAs (diseases) by their names. (TIF) [file pone.0087797.s002.tif]

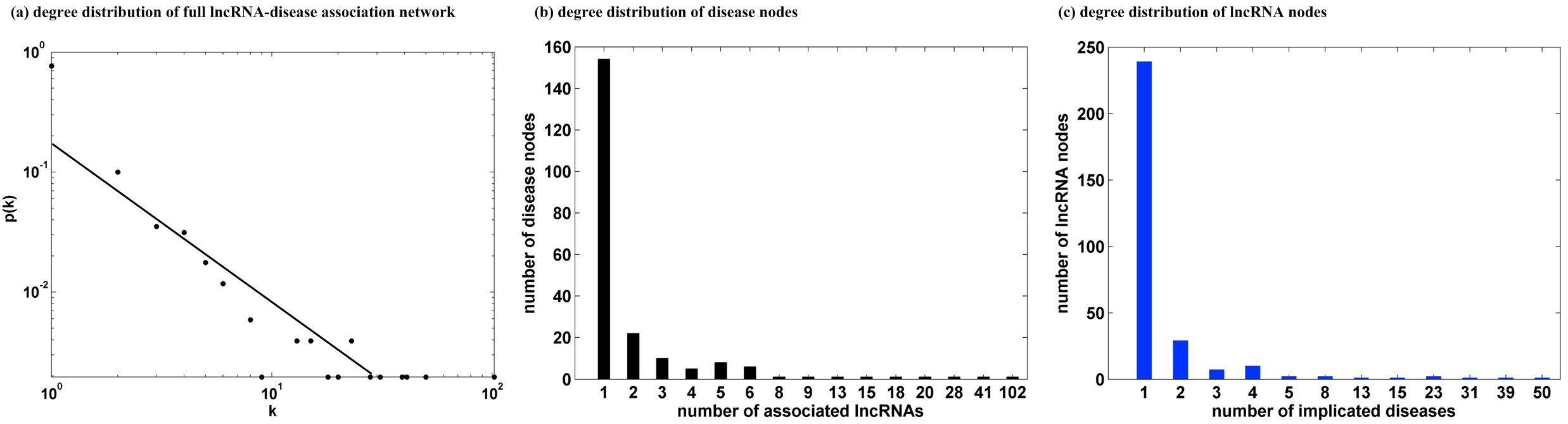

Supplement: Figure S2 — Degree distribution of full lncRNA-disease association network. (a) The degree distribution of the full lncRNA-disease association network. It closely follows a power-law distribution. Here, represents degree, denotes the fraction of nodes with a given degree . (b) Degree distribution of disease nodes in lncRNA-disease association network. (c) Degree distribution of lncRNA nodes in lncRNA-disease association network. (TIF) [file pone.0087797.s003.tif]

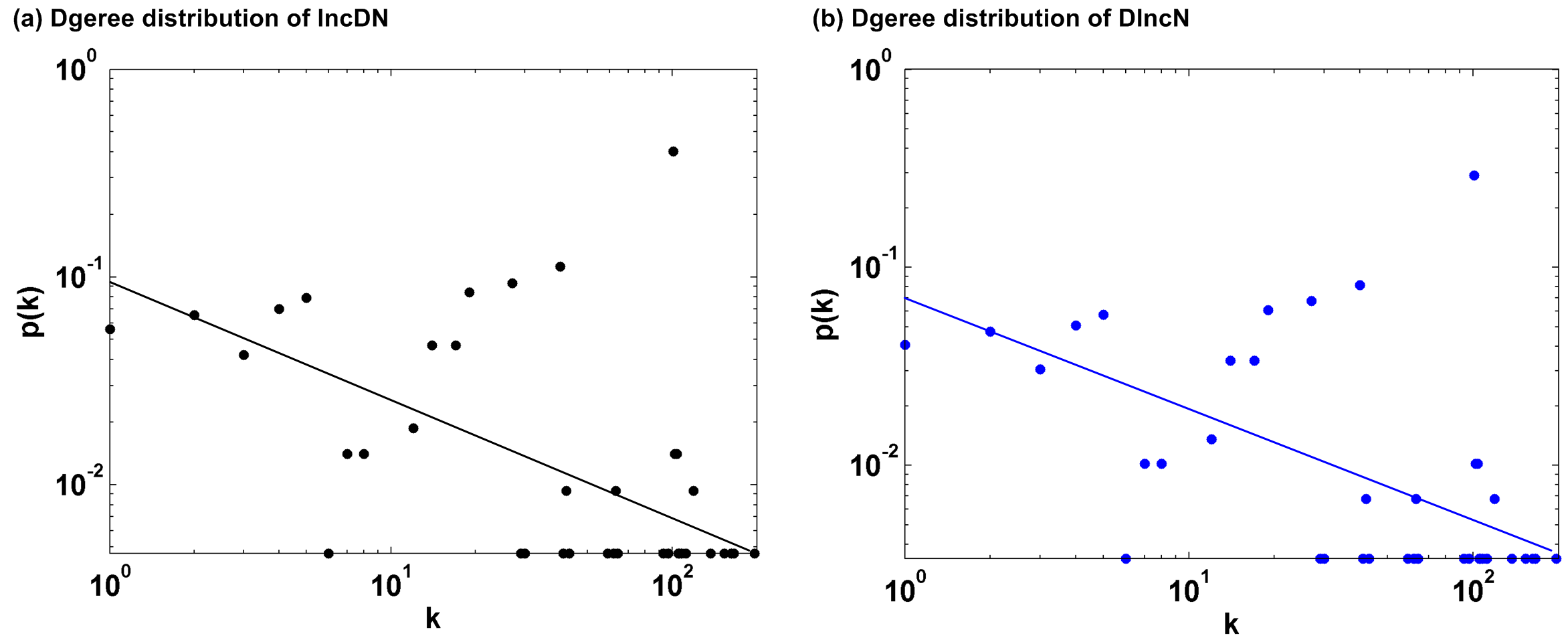

Supplement: Figure S3 — Degree distribution of lncDN and DlncN. (a) Degree distribution of lncDN. It closely follows a power-law distribution. Here, represents degree, denotes the fraction of nodes with a degree . (b) Degree distribution of DlncN. It closely follows a power-law distribution. Here, represents degree, denotes the fraction of nodes with a degree . (TIF) [file pone.0087797.s004.tif]

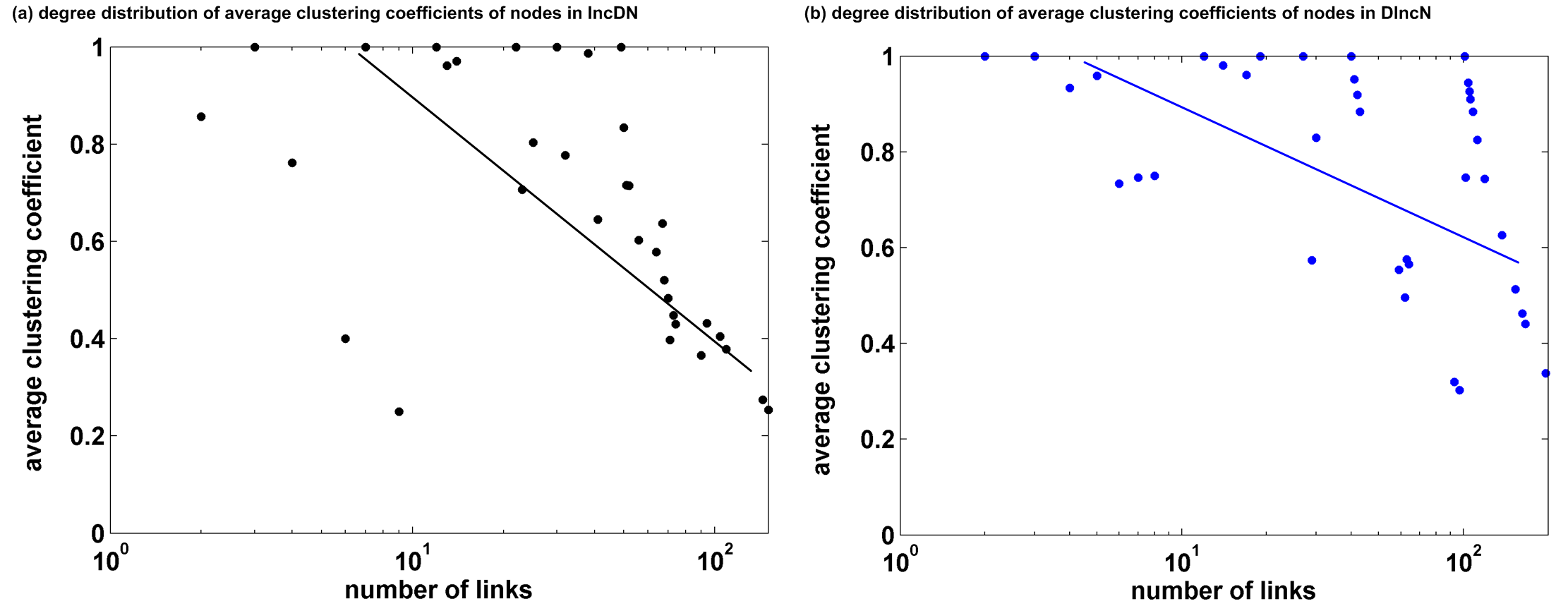

Supplement: Figure S4 — Degree distributions of average clustering coefficients of nodes in lncDN and DlncN. (a) Degree distribution of average clustering coefficients of nodes in lncDN. (b) Degree distribution of average clustering coefficients of nodes in DlncN. Both distributions are closely following a power-law distribution. (TIF) [file pone.0087797.s005.tif]

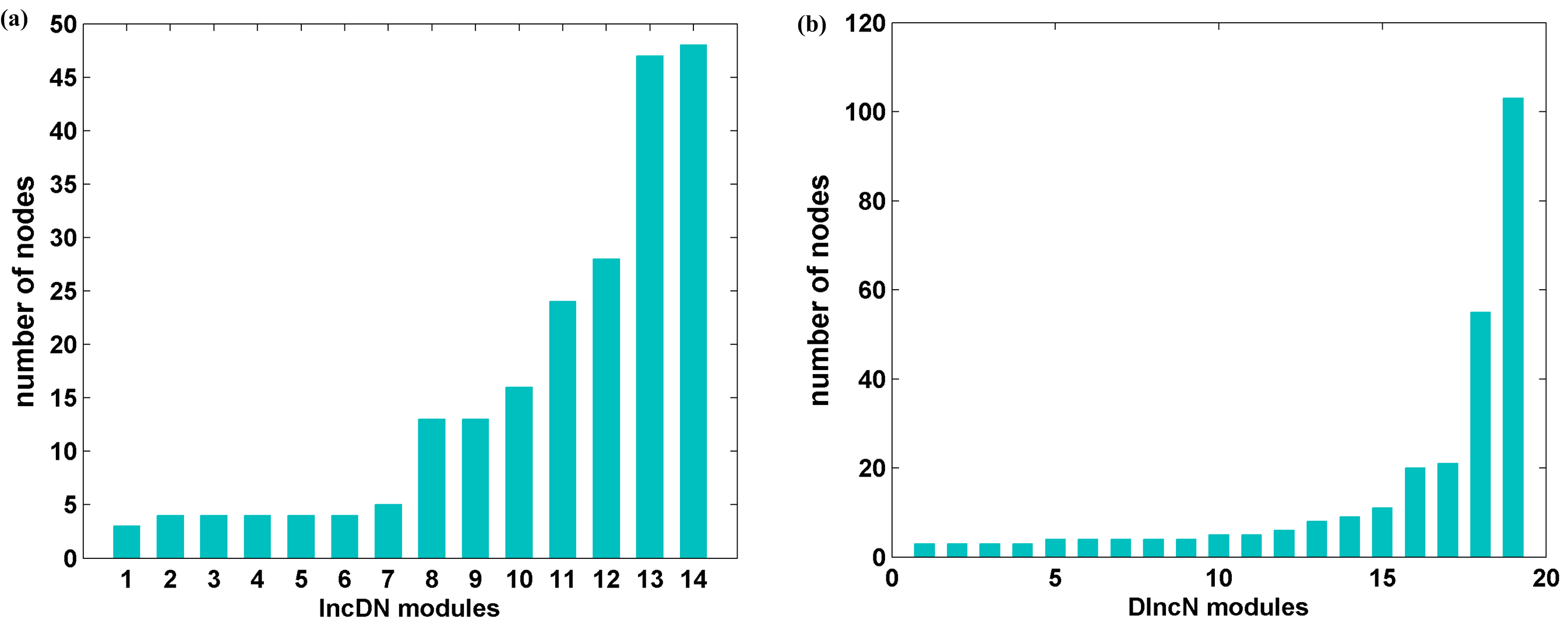

Supplement: Figure S5 — Distribution of module sizes in lncDN and DlncN. (a) The module sizes of 14 modules in lncDN. (b) The module sizes of 19 modules in DlncN. (TIF) [file pone.0087797.s006.tif]
